# The Certainties and Uncertainties of Tuberculosis

**Published:** 1908-02-01

**Authors:** 


### The Hospital

A Journal of

The Medical Sciences and Hospital Administration.

New Series. No. 46, Vol. II. [No. 1118, Vol. XLIII.] SATURDAY, FEBRUARY 1, 1908.

### THE CERTAINTIES AND UNCERTAINTIES OF TUBERCULOSIS.

The report "On Sanatoria for Consumption and certain other Aspects of the Tuberculosis Question," just issued by the Local Government Board, presents a forcible illustration of the extraordinary difficulty and complexity of many of the problems which necessarily engage the attention of the medical profession. Here is a disease which has long existed on a scale so extensive that every medical practitioner is thoroughly familiar with its ordinary clinical manifestations. For more than a quarter of a century the essential cause of the disease, the tubercle bacillus, has been known and recognised. In special institutions, and under favourable conditions generally, tuberculosis has been subjected to the most minute and searching investigations in its clinical, pathological, and bacteriological relationships. And last, but by no means least, the methods of experimental observation and research have been diligently employed with a view to unlock the secrets and mystery of the disease. Yet, in spite of all this, it remains true that to many questions which lie at the very heart of the problem no confident answer can even now be given. The discovery of the specific germ was, no doubt, an enormous step in advance, and on it, indeed, all modern views depend. But we are still uncertain of so elementary a fact as the route by which the bacillus usually gains access to the human body. The generally accepted view that infection occurs through the lungs is now open to serious doubt, as there is a considerable body of evidence that, even in cases of pulmonary tubercle, the avenue of approach is really the alimentary canal. Following on this comes the dispute as to the identity or non-identity of human and bovine tuberculosis, and the question whether in any material degree the disease is directly communicated from man to man. Still more vague and indefinite is that essential element of the problem which is suggested by such terms as immunity, tissue resistance, and family or personal predisposition. What, in other words, is the nature of that quality in the "soil" which in one case successfully resists the bacillus, and in another yields readily to the invader? These and similar questions have yet to be answered, and it is only when secure answers have been provided that a confident doctrine of tuberculosis can be formulated and a complete scheme of practical preventive measures can be devised.

The Local Government Board's report offers still other instances of the difficulties which attend the practical handling of the tuberculosis problem. One of these is found in the figures which present the behaviour of pulmonary tuberculosis in England and Wales during the last fifty years. That there has been a considerable decline in the mortality from this disease is well known. Thus in 1838 the deaths from consumption show an average of 39.9 in every 10,000 persons living; in 1851 the average had fallen to 27.7, and in 1906 to 11.5. The practical question, of course, is, What has been the cause of so marked and gratifying an improvement? But to find a sure answer to this question is difficult in the extreme. The favourable movement has been, with rare exceptions, continuous from year to year, and there has been no special acceleration of it immediately manifest after such events as the discovery of the bacillus in 1884, the institution of sanatoria, the public crusade against tuberculosis, or the increased power of municipalities to deal with questions of public health and with the housing of the working classes. In such circumstances it is impossible to avoid the doubt whether the steady decline of the disease may not be due to some force or influence of which we have no knowledge rather than to special efforts of a curative or prophylactic nature. Even if the latter be allowed the triumph, it still remains uncertain in what proportions the total credit must be divided amongst them.

Amidst many uncertainties, however, there are some positions which are beyond dispute. Thus it is now known that a healed or cured tuberculosis, so far from being an uncommon experience, is quite a common event. This, of course, is a very different view from the one which prevailed, say, thirty years ago, and its establishment has brought not only hope to the individual sufferer, but encouragement to attempts at rational treatment. Another decided gain in knowledge and practice is the recognition of the value of sanatorium discipline in the arrest of the disease in the individual victim. That this new departure was at first, as is common in such circumstances, stated in terms in excess of its merits cannot be denied, and it is still doubtful in what proportion of cases anything like sustained good results can be secured. But, without question, many patients have received a large measure of benefit, and —a not unimportant gain—there has been a general increase in the public appreciation of the value of hygienic measures. There is, further, good reason to hope that the careful study of consumptives collected in sanatoria has by no means exhausted the possibilities of the situation. Quite remarkable in this respect are the results reported by Dr. Marcus S. Paterson from the Brompton Hospital Sanatorium at Frimley. Dr. Paterson has made a new departure in the institution of a system of graduated labour tasks for consumptives, and the success attending this method is most impressive. More particularly is this success seen in the ability of patients so treated to return to, and to carry on, their usual occupations. Here, then, may be chronicled another advance in the effective treatment of a disease which until comparatively recent times was regarded as practically beyond control. It cannot be doubted that sooner or later the large problems to which we alluded at the outset will be satisfactorily solved. In the meantime, we must be content to acknowledge our ignorance on many points, but in doing so we are also justified in asserting that, in spite of great difficulties, our knowledge of tuberculosis and our capacity to deal with the disease are surely, even if slowly, advancing.

